# Navigation problems of ICSI or naive blastocyst can be solved with artificial blastocyst

**DOI:** 10.1186/s12958-018-0326-1

**Published:** 2018-01-30

**Authors:** Onder Celik, Mustafa Acet, Haldun Arpaci, Levent Dikbas, Aytac Imren, Bulent Duran, Nilufer Celik, Sudenaz Celik, Cihat Unlu, Ibrahim Sahin, Suleyman Aydin

**Affiliations:** 1 Private Clinic, Obstetrics and Gynecology, Usak, Turkey; 2 Private Clinic, Obstetrics and Gynecology, Istanbul, Turkey; 3 Department of Obstetrics and Gynecology, Kafkas University School of Medicine, Kars, Turkey; 4 Department of Obstetrics and Gynecology, Aksaray University School of Medicine, Aksaray, Turkey; 5 Department of Obstetrics and Gynecology, Medical Park Hospital, Usak, Turkey; 6 Adatip Private Hospital IVF Center, Kocaeli, Turkey; 7 Department of Biochemistry, BehcetUz Children’s Hospital, Izmir, Turkey; 8 Kent College Guzelbahce High School, Izmir, Turkey; 9 Acibadem University School of Medicine Department of Obstetrics and Gynecology, Istanbul, Turkey; 10 Department of Medical Biology Erzincan University, School of Medicine, Erzincan, Turkey; 11 Department of Medical Biochemistry (Firat Hormones Research Group), Firat University, School of Medicine, Elazig, Turkey

## Abstract

Embryos have evolved a remarkable capacity to find implantation site. The impressive navigation ability of natural blastocysts may rely on highly sensitive signals arising from embryos and specialized signal processing strategies in the endometrium. Navigation capabilities may be compromised in ICSI embryos because of altered biochemical signaling. The design and delivery of artificial blastocyst (AB) carrying strong chemical signals may allow ICSI embryos to more easily locate to and be retained in the implantation zone. ICSI embryos will attach easily to the implantation zone after it is found by the AB. Co-transfer of the AB together with the ICSI embryo may overcome potential difficulties in implantation due to impaired embryo-maternal communication in cases with implantation failure.

## Introduction

Less is known about how human blastocysts sense the spatial characteristics of their environment. The spatial orientation and navigation of natural/naïve blastocyst (NB) inside ampulla rely on information of landmarks associated with the endometrium. NB produces specific signals as a navigation system that is not exhibited by ICSI blastocyst (IB). Navigational abilities require knowledge of the ampullary location in relation to the endometrium. This biochemical knowledge is formed from blastocyst-derived signals and endometrial signals. More than half of embryos transferred in the assisted reproductive technologies (ART) cycle are not implanted. This failure may be due to either molecular, genetic, anatomical factors or embryo navigation problems. Most of the embryos transferred during the ART cycle are found in the vagina several hours after transfer suggests that these embryos may have navigation problems [1–5].

Artificial blastocyst (AB) equipped with powerful chemical signals are needed to solve the navigation problem of NB or IB. Replacement of receptivity molecules that mimic the effects of healthy blastocyst may enhance the implantation chance of poor quality embryos and may be a new treatment option. The delivery of AB and IB in the same catheter may allow genuine embryos to easily locate and be retained in the implantation zone. This paper builds on our clinical observations and isolated discussions of the navigation problems of naïve or ICSI embryos and the role of AB in triggering endometrium as well as improving navigation in women with implantation failure.

## How do natural blastocysts navigate to their final destination?

Developing embryos emerge from ampulla and begin trans-tubal journey as they reach the morula stage. Little is known about how bidirectional communication between the embryo and endometrium function. Signals from embryo or endometrium function as trans-tubal or trans-endometrial navigational markers and elicit changes in the rolling direction of embryos. The ability of embryos and the endometrium to produce signals as navigational markers is an adaptive mechanism that helps embryos avoid straying from their intended path, which is not suitable for implantation. Sub-endometrial or tubal contractions or ciliar activity contributes to the rolling movement of embryos towards to the tubal ostiums, where they enter the fundal region [1]. Embryos may derive positional information from the implantation site when they reach the endometrial cavity, which enables embryos to determine their position relative to the implantation site and change direction. One signal provides directional or compass information that enables embryos to maintain a course in a particular direction, such as up or down. Good quality embryos detect the implantation site with the help of signals and exploit these signals as a source of information for homing as well as to move to their final destination.

## How does NB prevent involuntary drift in other directions than dictated by receptivity genes?

One critically important aspect of embryo migration is the use of signal information to identify and remain in the implantation zone. Another equally important migratory task is the determination of when and/or where the journey should end. A small deviation in time and place may lead the embryo to go a different region and result in death. NB may use different forms of chemical and physical signals as a compass to choose and attach to a desired course. NB detects and responds to signals from decidua to compensate for involuntary drift and correctly find their destination. NB may also possess a map sense to determine its anatomical location in the tuba uterina or endometrium suggesting NB is capable of “true navigation”. The bidirectional chemical signals are highly important for controlling embryo migration, but it is not unreasonable to imagine that the physical properties of the fallopian tubes and endometrium which are used to maintain the desired voyage direction [6]. The current shape of the endometrial cavity, decidual pattern, expression quality of receptivity genes and endometrial volume are obvious compass cues to compensate for involuntary drift. However, NB should have a “preferred inherited direction” to maintain this direction and achieve a common orientation in the dark and narrow area of the fallopian tubes and endometrium. The increased incidence of implantation failure and the existence of genetically abnormal embryos in aging women support the hypothesis that the genetic properties of the endometrium or embryo somehow influence the bidirectional navigational system. The migration of the IB is less well understood than its naïve counterpart. Different from NB, IB use chemical cues that are much less reliable to identify the target point. IB has also lower implantation rates than NB [7]. Enhanced pregnancy rates following oocyte donation suggest that young IB may possess a map sense and true navigation.

## Are the navigation behaviors of NB and IB identical?

The navigation behavior of IB is different from that of NB for the following reasons. (i) The entry speed of NB into the uterine cavity is different from that of IB. The negative uterine pressure and tubal contraction determine the NB’s entry speed. (ii) The medium type and quantity as well as the pressure applied to the transfer catheter determine the IB’s entry speed into the endometrium. (iii) Defects in the shape or volume of the endometrium or fallopian tubes may adversely affect the embryo’s entry speed into the endometrial cavity. (iv) Trans-cervical passage of the transfer catheter may adversely affect negative uterine pressure in connection with implantation. (v) The air inside the transfer catheter may degrade the communication between the embryo and receptive zone. The polyvinylpyrrolidone inside the transfer catheter may degrade the reliability of chemical cues for navigation. (vi) Multiple egg development and deactivation of the tubes in the ART cycle may adversely affect the signal communication between the embryo and endometrium [7].

## What is artificial blastocyst?

Ovarian and endometrial aging are the main causes of implantation failure [8]. Few options are available to improve endometrial receptivity or oocyte quality. The success rates of oocyte donation supports the role of young oocytes on implantation [9]. Good quality euploid embryos attach to any abdominal tissue in which the impact of estrogen may be observed. However, further development of embryos requires the existence of endometrium. Oocyte donation is not possible for every woman who suffers implantation failure. Therefore, focusing our attention on the endometrium trigger to improve receptivity remains as an essential step for treatment of implantation failure [10, 11]. A sufficient quantity receptivity gene has to be expressed by the endometrium for implantation. However, many patients cannot achieve this and navigation capabilities may be compromised in ICSI embryo because of altered biochemical signaling. Synthetic beads coated with receptivity molecules exhibit well characterized decidual response similar to that produced by genuine blastocyst [11]. We defined AB as a synthetic or physiological container coated with implantation-promoting chemo-attractants. AB may allow for endometrium triggering and the releasing of receptivity molecules. Co-transfer of AB and IB make possible ICSI embryos to more easily locate to and be retained in the implantation zone.

## The design and development of AB

We can perform our artificial blastocyst hypothesis in three stages. (i) The first stage is the design phase of the containers. During this phase the design and development of synthetic containers having the same size of a human blastocyst are necessary. Containers should be biodegradable molecules of synthetic origin or immature eggs collected during oocyte pick up. Metaphase I, II or germinal vesicle stage oocytes or empty zona can be used as physiological containers. Synthetic containers may be in the form of nano-particles or melting beads. (ii) The second phase is the loading phase of the containers. Basic receptivity molecules such as bone morphogenetic protein 2, transforming growth factor beta 1, Activin-A, the gene encoding heparin binding EGF-like growth factor, insulin-like growth factor-1 and -2, cyclic adenosine monophosphate, cyclooxygenase 2, homeobox genes, leukemia inhibitory factor, IL-6, IL-11, integrins, and e-selectin are the first choice for loading of containers [10–12]. (iii) The third and final phase is the transfer phase of the containers.

## Intrauterine transfer time and methods of AB

The AB can be transferred to the endometrial cavity alone or together with IB on the day of embryo transfer. However, the transfer of AB before the transfer of IB may save time for the endometrium to become receptive. AB can be transferred into the endometrium via intrauterine injection or conventional embryo transfer methods.

## The impact of AB on epigenetic of IB

The transfer of synthetic containers may lead to abnormal decidual swelling or implantation of the containers. This may cause epigenetic changes in IB. To prevent implantation of containers we should use programmed beads which undergo lysis after the transfer. However, as the oocyte and the sperm are being exposed to several physical manipulations during IVF, the possible epigenetic alterations in IB due to AB can be ignored.

## Conclusions

Embryo must emit numerous signals and perceive signals from the endometrium to identify the implantation zone. However, many NB or IB that appears to be morphologically healthy they cannot produce sufficient signals to find the implantation zone. Increased decidua formation and implantation rates in multiple embryo transfer cycles have led to the hypothesis that the implantation zone can be found more easily with the use of AB. Co-transfer of AB and IB may allow secretion of different types of cytokines and growth factors, which are required for decidualization [5, 10, 11]. Significant increases in decidualization were reported at blastocyst-implantation cycles [2–5], suggesting that AB may induce the receptive zone through the secreted signals. Transient attachment of AB to the endometrium may also initiate and promote decidualization physically.

Finally, we can prefer to use AB for the following purposes: (i) regulation of the expression of receptivity genes and molecules associated with implantation; (ii) mechanical or chemical induction of decidualization; and (iii) regulation of blastocyst adhesion and attachment. Co-transfer of an AB together with IBs may overcome potential difficulties in implantation due to impaired embryo-maternal communication in patients with implantation failure.

## Abbreviations

AB: Artificial blastocyst
ART: Assisted reptoductive technology
IB: ICSI blastocyst
ICSI: Intracytoplasmic injection
IVF: In vitro fertilization
NB: Naïve/natural blastocyst
